# From Placenta to Polycystic Ovarian Syndrome: The Role of Adipokines

**DOI:** 10.1155/2016/4981916

**Published:** 2016-09-26

**Authors:** Chiara Sartori, Pietro Lazzeroni, Silvia Merli, Viviana Dora Patianna, Francesca Viaroli, Francesca Cirillo, Sergio Amarri, Maria Elisabeth Street

**Affiliations:** ^1^Department of Paediatrics, Arcispedale Santa Maria Nuova-IRCCS, Reggio Emilia, Italy; ^2^Department of Paediatrics, University Hospital of Parma, Parma, Italy

## Abstract

Adipokines are cytokines produced mainly by adipose tissue, besides many other tissues such as placenta, ovaries, peripheral-blood mononuclear cells, liver, muscle, kidney, heart, and bone marrow. Adipokines play a significant role in the metabolic syndrome and in cardiovascular diseases, have implications in regulating insulin sensitivity and inflammation, and have significant effects on growth and reproductive function. The objective of this review was to analyze the functions known today of adiponectin, leptin, resistin, and visfatin from placenta throughout childhood and adolescence. It is well known now that their serum concentrations during pregnancy and lactation have long-term effects beyond the fetus and newborn. With regard to puberty, adipokines are involved in the regulation of the relationship between nutritional status and normal physiology or disorders of puberty and altered gonadal function, as, for example, premature pubarche and polycystic ovarian syndrome (PCOS). Cytokines are involved in the maturation of oocytes and in the regular progression of puberty and pregnancy.

## 1. Introduction

Adipokines are produced mainly by adipose tissue, a dynamic endocrine organ involved in the regulation of various endocrine processes such as glucose and fatty-acid metabolism, energy expenditure, inflammatory response, cardiovascular function, and reproduction [1, 2]. In recent years, adipokines have been found to be produced by other organs also, that is, placenta, ovaries, peripheral-blood mononuclear cells, liver, muscle, kidney, heart, and bone marrow [3, 4], and have been related mainly to insulin sensitivity, inflammation, and other functions [5–7].

Insulin sensitivity and inflammation have been shown to influence both growth [8–10] and ovarian function; in particular there is wide evidence that polycystic ovarian syndrome (PCOS) originates from insulin resistance [11–14] and from excessive exposure to insulin. Excessive exposure to insulin is related to overgrowth and skin lesions, such as acanthosis nigricans, and changes in adipokines, including reduced adiponectin and leptin levels [1, 3].

Interestingly, certain conditions such as being born small for gestational age are associated with an increased risk of insulin resistance, metabolic syndrome, and poor growth [15, 16]. Since Barker's theory on the fetal origin of adult disease, it has become clear that the programming of endocrine and metabolic systems is set in utero.

In this review we shall focus mainly on adiponectin, leptin, resistin, and visfatin whose functions are to date better studied compared to other adipokines, considering findings in placenta and newborns, and further described knowledge related to childhood and adolescence.

## 2. Description of the Adipokines under Examination

### 2.1. Adiponectin

Adiponectin is a 30 kDa secretory protein, which is produced mainly by adipocytes, but also by the placenta, osteoblasts, and cardiomyocytes [8, 17–20].

It is expressed as a full-length protein or as a smaller and globular fragment; this latter form is generated by proteolytic cleavage by leukocyte elastase secreted by activated monocytes and neutrophils. Adiponectin circulates as multimers of high, middle, and low molecular weight.

The low-molecular-weight isoforms and trimeric isoforms seem to have effects on the central nervous system [21].

Adiponectin receptors (AdipoR1 and AdipoR2) are integral membrane proteins that are ubiquitously expressed, mediating adiponectin's function at both the central and peripheral levels.

The action mediated by AdipoR1 is for globular adiponectin, while AdipoR2 is a receptor for full-length adiponectin. In cultured cells, such as myocytes and hepatocytes, these receptors increase AMP kinase (AMPK) activities, peroxisome proliferator-activated receptor alpha (PPAR-alfa) activities, fatty-acid oxidation, and glucose uptake [22, 23].

Suppression of AdipoR1 reduces fatty-acid oxidation, which is mediated by globular adiponectin. Suppression of AdipoR2 increases fatty-acid oxidation which is mediated by full-length adiponectin. Recent studies show that knockout AdipoRs mice exhibit insulin resistance [24].

In addition, AdipoRs are also decreased in obesity; several authors have demonstrated that expression of both AdipoR1 and AdipoR2 are significantly decreased in muscle and adipose tissue of insulin-resistant mice [25].

These data suggest that adiponectin receptors AdipoR1 and R2 play important roles also in the regulation of insulin sensitivity and glucose metabolism [26].

The main action of adiponectin consists in an upregulation of insulin and energy balance [27]. Functional and genetic studies on adiponectin strongly suggest that reduction in adiponectin levels plays a causal role in the development of insulin resistance (IR), metabolic syndrome, type 2 diabetes, and atherosclerosis. Plasma adiponectin levels are decreased in obesity. In the skeletal muscle, adipokines increase expression of fatty-acid transport and, by activation of peroxisome proliferator-activated receptors (PPARs), increase fatty-acid combustion and energy consumption [28]. Moreover, adiponectin enhances insulin secretion to inhibit gluconeogenesis in hepatocytes [29]. Reductions in plasma adiponectin levels are commonly observed in a variety of states frequently associated with insulin resistance and metabolic syndrome as shown by von Frankenberg et al. who demonstrated that adiponectin levels decrease by increasing the number of metabolic syndrome criteria [5].

Obesity is well known to be characterized by a state of low grade chronic inflammation. Interestingly, adiponectin has been shown to have an anti-inflammatory action and is reduced in obesity. Adiponectin primarily acts modulating macrophage function suppressing TNF-alpha secretion by inhibiting TNF-alpha transcription [30]. This inhibitory action plays an important role on NF-kb activation mediated by TNF-alpha [30, 31]. Adiponectin stimulates also IL-10 and IL-1 receptor antagonist (RA) production. It is well known that IL-10 has an anti-inflammatory action and can inhibit production of many proinflammatory cytokines [32, 33]. Summarizing, low adiponectin in obesity may contribute to explaining inflammation in these patients.

### 2.2. Leptin

Leptin is the 167-amino acid product of the human leptin gene (ob gene). It is primarily secreted by the white adipose tissue but is also produced by placenta [34]. Leptin secretion is pulsatile and has significant diurnal variations with higher levels in the evening and early morning and shows relationships with other hormones having a circadian rhythm such as cortisol. The relationship between these two hormones is explained by the direct stimulatory action that leptin has on CRH [35].

Leptin effects are mediated by binding to specific leptin receptors, expressed in the brain and in peripheral tissues such as muscle, liver, adipose tissue, and so forth. There are several isoforms of leptin receptors (ObRs), the most important are ObRa and ObRb [36].

Different functions of leptin have been described, including mediation of food intake, liver glucose production, gonadotropin secretion, suppression of lipogenesis in adipose tissue, and modulation of immune response [37, 38]. Circulating levels of leptin are related to adipose mass. Leptin levels decrease consistently during starvation: low leptin levels induce complex mechanisms in order to preserve energy, that is, increase in appetite, decrease in thermogenesis, decrease in locomotor activity, inhibiting the hypothalamus-pituitary-thyroid axis, activating the adrenal axis, and inhibiting reproductive function [37]. Leptin levels are related to timing of GnRH and LH secretion as shown in some studies that speculate that reduced pulsatility of LH during the night may be related to low leptin levels present during these hours [39, 40].

The absence of leptin or mutations in its receptor induce obesity and hyperphagia. Leptin deficient humans are obese, diabetic, and infertile [41]. This adipokine may therefore play a critical role in regulating both energy homeostasis and the reproductive system, acting as a signal of energy reserve essential for normal reproductive function.

### 2.3. Resistin

Resistin is a 12.5 kDa peptide, whose name derives from its ability to resist insulin action. In humans, this adipokine is mainly secreted by peripheral-blood mononuclear cells. There are two different forms of resistin: low- and high-molecular-weight isoforms [8, 42]. Resistin acts through interaction with Toll-like receptor (TLR) 4 in human myeloid and epithelial cells. TLR activation initiates a cascade of intracellular events leading to alterations in several transcriptional and signaling pathways, including NF-kb signaling, and thus is tightly connected with inflammatory responses [43].

Resistin was first described as a factor contributing to the development of insulin resistance and diabetes mellitus in humans; a debate is still ongoing regarding its exact role in obesity, in insulin sensitivity, and in the development of type 2 diabetes mellitus (DM2) [44]. Resistin may represent a link between inflammatory processes and metabolic signals. Circulating levels of resistin are increased in obesity. Many authors indicate a solid association between this adipokine and insulin resistance [45, 46]. Some data suggest that resistin has a role in inflammatory processes, and resistin seems to directly cause endothelial dysfunction [47]. Resistin stimulates secretion of proinflammatory cytokines such as TNF-alpha and IL-6 by macrophages through the NF-kb pathway [48].

In mouse and human models, resistin levels increase in severe inflammatory diseases. An increase in resistin levels has been described in patients having nonalcoholic fatty liver disease (NAFLD) [49], and resistin serum levels have been reported to correlate with hepatic inflammation and necrosis [50].

### 2.4. Visfatin

Visfatin is a 491-amino acid adipokine. It was previously identified as a protein involved in B cell maturation and is identical to pre-B cell colony-enhancing factor (PBEF), described in 1994 as a cytokine produced by lymphocytes, acting on lymphocyte maturation and inflammatory regulation [51]. Visfatin acts both as a paracrine and an autocrine agent and is secreted by adipose tissue, but it seems to be expressed also in liver, muscle, kidney, heart, bone marrow, and trophoblast and fetal membranes [52, 53].

Recently, visfatin was described as a protein with insulin-like functions. In human studies, a positive correlation between visceral adipose tissue and visfatin gene expression was reported, along with a negative correlation between visfatin and subcutaneous fat, suggesting a different regulation of its expression in these different depots. High levels of visfatin were found in visceral and omental adipose tissue. In contrast, subcutaneous adipose tissue expressed low levels of visfatin [54]. Variable results have been obtained regarding the relationship between visfatin and diabetes or insulin resistance with some authors showing increased visfatin levels in type-2 diabetes and PCOS patients [55, 56].

Furthermore, the role of visfatin in ovarian and/or uterine regulation, via insulin sensitivity or through other mechanisms, remains to be demonstrated yet [2].

In Table 1 we summarize the site of production and the relationship of the above-mentioned adipokines with inflammation, metabolic and reproductive functions.

## 3. Role of Adipokines in Pregnancy, Placenta, and Fetus

The above-mentioned cytokines have all been described in the human placenta in recent years and shown to be related somehow to fetal growth and adiposity [57]. In recent years a potential importance of epigenetic changes in some adipokines is arising relative to metabolic programming and might be involved in the regulation of subsequent postnatal growth and puberty [58].

### 3.1. Adiponectin

Adiponectin is a placental-produced adipokine whose concentrations have been shown to be related to birth weight [8, 59].

Adiponectin concentrations in cord blood of at-term neonates are two to three times higher than those reported in adults [60–62]. The mechanisms by which adiponectin levels are regulated in the placental tissue are not yet well defined. In adults, an increase in fat mass is associated with increasing adiponectin levels, whereas weight loss is associated with reduction in adiponectin concentrations. Moreover, fat mass seems to act as a negative feedback for adiponectin secretion; the lack of such negative feedback in newborns could contribute to hyperadiponectinemia.

Several authors showed a positive correlation between cord blood adiponectin concentrations and birth weight, with higher adiponectin levels in adequate for gestational age (AGA) newborns compared to small-for-gestational age (SGA) and intrauterine growth restricted (IUGR) newborns [63–65]. Adiponectin in placental lysates has been described to be significantly lower in IUGR compared with AGA newborns and positively correlated with the weight of the placenta, birth weight, and head circumference [8].

In this respect, a study from Mazaki-Tovi et al. [66] evaluated adiponectin and leptin levels in twins with discordant and concordant growth: in the discordant twin group, while there was no significant difference in the median cord blood leptin concentrations between SGA twins and AGA cotwins, adiponectin levels were significantly lower in growth restricted newborns compared with the concentrations observed in the concordant twins which were similar.

Adiponectin acts as an insulin sensitizing hormone reducing hepatic glucose production and enhancing the insulin-mediated actions of the liver. Low adiponectin levels in low birth weight newborns may therefore predict the subsequent development of visceral fat and insulin resistance.

Moreover, Cianfarani et al. [65] evidenced particularly lower adiponectin levels in SGA children who showed postnatal catch-up growth, compared to those who remained small during late childhood, suggesting a potential role of early catch-up growth for subsequent development of metabolic disturbances.

Regarding large for gestational age (LGA) newborns, another study from Mazaki-Tovi et al. showed that adiponectin placental and cord blood concentrations were lower in macrosomic newborns than in the AGA counterparts [63].

Given that fat mass acts as a negative feedback on adiponectin production and given that hypertrophic adipocytes produce less adiponectin, we can consider the low expression of adiponectin in LGA as a manifestation of these assumptions.

As mentioned above, adiponectin is known to act as an insulin sensitizer. Recent studies showed a positive association between adiponectin and birth length, which may be the expression of the insulin sensitizing action of adiponectin on tissues [64]. Furthermore, Oshima et al. [67] demonstrated an adiponectin-induced activation of osteoblasts, an action that is likely linked to fetal linear growth.

In this context, Ibáñez et al. [68] recently described that IUGR fetuses show a different pattern of adiponectin production, shifted to the high-molecular-weight isoform which is known to be the most closely related isoform to insulin sensitivity. We can assume that this different pattern acts as an insulin sensitizer in the fetus and may promote fetal catch-up growth.

Another aspect of the expression of this adipokine, which suggests a role for adiponectin in fetal growth, is the inverse correlation described between its serum levels and gestational diabetes or obesity [64].

Interestingly, adiponectin gene DNA methylation has been shown to be influenced by maternal hyperglycemia [58, 69]. Bouchard et al. described lower methylation in the promoter of the adiponectin gene on the fetal side of the placenta in the presence of maternal hyperglycaemia, suggesting that this could contribute to the increased risk of reduced insulin sensitivity, obesity, and diabetes in the offspring in postnatal life [69].

### 3.2. Leptin

Expression of leptin is widespread in fetal and placental tissues [70] and a relationship between umbilical leptin concentrations and fetal growth indexes, such as birth weight, length and head circumference, and bone mineral density, has been established [71, 72]. Leptin serum concentrations are elevated in the fetus at term, probably acting as a feedback modulator of nutritional conditions and substrate supply [71].

A significant correlation between leptin levels in umbilical cord blood and birth weight of neonates has been described [71, 72]. Infants born small for gestational age (SGA) show lower leptin levels than their appropriate for gestational age (AGA) counterparts [73].

Lea et al. [74] described a relationship between low concentrations of leptin and fetuses born at term with intrauterine growth restriction (IUGR), whereas macrosomic offspring of diabetic mothers showed high leptin serum concentrations. This latter finding was thought to be possibly related to maternal hyperglycaemia that leads to fetal hyperinsulinaemia, which in turn would cause macrosomia via elevated leptin serum levels. In the same study, the placental expression of leptin in a twin pregnancy in which one baby was normal sized while the other showed growth retardation was evaluated: lower levels of leptin were detected in the samples of the growth retarded twin. Tsai et al. [75] and Vela-Huerta et al. [76] found significantly higher leptin levels in large for gestational age (LGA) infants than in their AGA counterparts.

Furthermore, leptin has been repeatedly found to be positively related to other factors (i.e., insulin and IGF-I) which are well known to be associated with intrauterine growth.

New insights into the role of leptin in fetal growth have shown cell and tissue specificity, thus leading to speculate that its role in fetal growth may be cell- and tissue-specific [73–75].

Mise et al. [77] investigated the changes in maternal leptin levels in pregnancies complicated by preeclampsia: maternal plasma leptin levels were significantly higher than those of the control group and showed a negative correlation with neonatal body weight. Furthermore, leptin mRNA expression was significantly increased in placenta from women with pre-eclampsia compared to the control group, showing a similar behaviour as other inflammatory cytokines in that condition. Plasma leptin levels in affected women who delivered SGA newborns were significantly higher than those in women who delivered AGA newborns [78]. Furthermore, the incidence of SGA newborns in women who showed elevated plasma leptin levels was higher than that in women with normal leptin levels. These findings suggested a close correlation between fetal growth restriction and elevated maternal plasma leptin levels [78].

A study comparing pregnancies complicated by gestational diabetes with macrosomic babies and healthy age-matched pregnant women and their newborns concluded that gestational diabetes was associated with a downregulation of maternal Th1 cytokines (IL2 and IFN gamma) and an upregulation of leptin and inflammatory cytokines [79]. Furthermore, during pregnancy characterized by impaired glucose tolerance and gestational diabetes, placental DNA methylation was reported to be increased on the maternal side of the placenta and to be related to hyperglycaemia. This was associated with leptin gene methylation in placenta and with increased circulating leptin levels in hyperglycaemic pregnant women [80]. These mechanisms could further confirm why newborns exposed to gestational diabetes mellitus have an increased risk of developing obesity and T2DM [58, 81].

### 3.3. Resistin

Yura et al. [82] and Lappas et al. [83] showed that resistin is expressed in the human placenta. Yura et al. measured resistin expression in maternal adipose tissue, placenta, and fetal membranes and they found out that trophoblastic resistin gene expression was significantly higher in at term placenta than in first trimester placental tissue and that plasma resistin levels in pregnant women were higher than resistin levels in nonpregnant women [82]. High concentrations of resistin in cord blood samples may suggest that this hormone is potentially related to the control of fetal energy expenditure and deposition of adipose tissue.

The expression of resistin in the human placenta is higher than that in adipose tissue. It has been hypothesized that placental resistin may have a physiological meaning in the regulation of maternal glucose metabolism by decreasing insulin sensitivity during human pregnancy. Resistin in placental lysates has been reported to be positively correlated with placental insulin concentration [8].

In the study from Lappas et al. resistin levels were found to be significantly lower in macrosomic fetuses of diabetic mothers compared to control and to be higher in growth restricted pregnancies compared to normal [83]. Maternal and cord blood serum resistin seemed therefore to have a negative correlation with birth weight. Resistin is likely to have an inhibitory effect on adipose conversion, acting as a feedback regulator of adipogenesis. In this respect, as to the fetus, one could assume that low resistin levels in serum are related to excessive production of adipose tissue. This idea could explain the negative correlation described between umbilical resistin concentrations and birth weight.

However, Savino et al. [84], interestingly, found no correlation between breast milk resistin concentrations and maternal anthropometric measurements.

Another study from Vitoratos et al. [85] did not show significant differences in umbilical resistin levels between infants born from women with gestational diabetes (GMD) as compared to normal pregnant women.

In a recent review of the literature [86], according to the data from the 11 studies analyzed, there was no association between circulating resistin levels and gestational diabetes.

So far, we therefore do not have univocal results regarding the role of this adipokine in determining fetal growth and metabolism in normal and abnormal pregnancies.

### 3.4. Visfatin

Findings concerning visfatin and its possible relationships with fetal growth are controversial and often inconsistent despite recent findings of high concentrations of visfatin in cord blood which leads to assuming a placental production and relationships with fetal growth. Higher visfatin levels have been found in term IUGR newborns compared to their AGA counterparts. This finding may be linked to the different visceral adiposity or altered visceral adiposity in IUGR, which is known to be related to insulin resistance in adulthood [87].

Hu et al. [88] reported that serum visfatin levels were markedly decreased in women with pre-eclampsia compared with normal pregnant women. These findings suggested a role of visfatin in the determinism of this condition. Moreover, the same study showed no difference in BMI between affected and nonaffected women, suggesting that pathophysiology of pre-eclampsia and visfatin levels are BMI-independent.

In Table 2 we summarize the role of the above-mentioned adipokines in the determinism of fetal and postnatal growth and in the onset of gestational metabolic disturbances such as gestational diabetes.

## 4. Role of Adipokines in Puberty

Birth weight, insulin sensitivity, and adipokines are involved in puberty and its disorders. Puberty is a complex physiological process influenced by different factors, including neuroendocrine circuits and many circulating molecules, some of which still remain unknown. Many authors report a relationship between increasing rates of overweight and obesity in childhood and a trend towards an anticipation of pubertal onset, especially in girls. These observations have generated an increasing interest in pinpointing the mechanisms by which pubertal development and reproductive function are influenced by nutritional status. Pubertal development is physiologically characterized by an increase in adipose mass and changes in adipose tissue distribution. Moreover, several authors reported an increase in serum adipokine concentrations. These biochemical and metabolic modifications seem to be closely associated with the status of insulin resistance observed during this period. Adipokines represent an important link between nutritional status and pubertal physiology and several pubertal disorders, such as premature adrenarche, premature pubarche, polycystic ovarian syndrome, and constitutional delay in growth and puberty.

### 4.1. Puberty in SGA and AGA Newborns

Fetal programming of the endocrine axis related to intrauterine growth and events occurred during pregnancy may also contribute to the timing of puberty and to the future reproductive capacity. Pubertal development disorders influence not only sexual maturity, but also adult height, bone mineral density, and reproductive health [89].

It is well known that many factors such as midparental height, chronic diseases, medications, and pubertal development influence final height. Being born SGA according to either weight or length is a risk factor for growth and development disorders, as it is described that they are at higher risk of presenting a final height below their midparental height, as well as developing cardiovascular disease, obesity, diabetes, and other metabolic disorders [89].

Postnatal growth of SGA children has been analyzed in many studies [90–93].

The mean adult height of individuals born SGA was estimated to be approximately 1 SD lower compared to those born appropriate for gestational age (AGA). Furthermore, adult height is lower in subjects born short for gestational age than in those born light for gestational age [90, 93].

Most studies on pubertal development in children have explored the relationship between precocious puberty and pubertal growth in children born SGA. Precocious puberty causes advanced bone maturation, accelerated closing of epiphysis, and may compromise adult height. Precocious pubarche and precocious adrenarche have been shown to be more frequent in subjects with a low birth weight [94, 95].

Puberty is associated with a physiological state of insulin resistance, and conditions as premature pubarche have also been related to reduced insulin sensitivity and shown to be more frequent in subjects born SGA [96, 97].

Furthermore, girls with previous prenatal growth restriction have been described to have more frequently than the general population idiopathic functional ovarian hyperandrogenism and hyperinsulinism at 18 years of age [95].

Neville and Walker [96], in a retrospective Australian study of 89 children with precocious pubarche, also reported that being born SGA according to weight and/or length was an independent risk factor for precocious pubarche, along with prematurity and being overweight/obese. Moreover, SGA children with precocious pubarche show greater changes in weight SDS than AGA children.

It was suggested that rapid weight gain in childhood might predispose to precocious pubarche in susceptible individuals.

Most authors agree on the existence of a relationship between being born SGA, premature pubarche, and exaggerated precocious adrenarche. The possible causes of this association are thought to be insulin insensitivity, increased central adiposity, increased IGF-I levels between the ages of 2 and 4 years and metabolic, and hormonal patterns that are common in SGA children with excess weight gain in early childhood. High levels of IGF-I and insulin resistance stimulate adrenal androgen secretion and the development of precocious pubarche [94, 95, 97].

Several studies focused on timing and progression of puberty in children born SGA, but the results are difficult to compare due to variations in the definition of SGA, criteria of inclusion, and follow-up periods.

In a large population study on postnatal growth, 87% of SGA children showed full catch-up growth and reached puberty at a normal age. SGA children who remained short reached puberty earlier than those who presented catch-up growth [92, 98].

Persson et al. studied puberty in children divided according to perinatal risk factors: SGA, large for gestational age, short for gestational age, long for gestational age, born prematurely from a pregnancy complicated by pre-eclampsia, and they also considered a group of children without perinatal risk factors. The onset of puberty was defined according to the age at growth spurt. Interestingly, in boys the mean age at onset of puberty was similar based on the presence or absence of risk factors, whereas in girls the onset of puberty was earlier in those born short or light for gestational age [99].

Interestingly, Lazar et al. reported that both SGA children with short stature and AGA children with idiopathic short stature enter puberty at a normal age, but SGA children show a distinct pubertal growth pattern compared to AGA [15].

There is no general agreement on the existence of differences in age at menarche between SGA and AGA girls. Several longitudinal follow-up studies do not find significant differences in the progression of puberty or age at menarche between girls born SGA and AGA [91, 100] while other studies report an earlier age of menarche in girls with fetal growth restriction compared to girls born of appropriate birth weight [95, 101].

Interestingly, one study evaluated the development of premature AGA and full-term SGA children, observing that menarche occurred 6 months earlier in the preterm group and 12 months earlier in the SGA group with respect to full-term AGA controls. The interval of time between onset of puberty and menarche was similar in all groups [102].

If premature pubarche occurs, then menarche before the age of 12 has been reported to be 3 times more prevalent among girls born SGA compared with girls with normal birth weight [95, 97].

## 5. Adipokines and Pubertal Development

### 5.1. Adiponectin

Many studies have shown, in animal models, that adiponectin concentrations change throughout pubertal maturation [103]. Human data confirm this trend and show differences in the pattern of secretion during puberty between males and females. In healthy lean males, but not in females, adiponectin concentrations significantly decline during pubertal maturation, with lower adiponectin levels being observed in adolescent boys compared with girls [104]. The authors of this study further showed a negative correlation between adiponectin concentrations, testosterone, and dehydroepiandrosterone sulfate serum levels; however, pubertal stage was shown to be the strongest independent predictor of adiponectin concentrations, followed by body mass index and testosterone. This model did not seem to fit for female pubertal development. Experimental data have shown for testosterone, but not for estrogens, an inhibitory action on adiponectin secretion from adipocytes, confirming a closer correlation between androgens and adiponectin compared with estrogens [105].

Data like these may well explain the gender differences reported for this adipokine concentrations in adults, with the lowest adiponectin levels being found in males [104].

According to recent studies focused on female reproductive function, although adiponectin concentrations do not seem to change significantly during the menstrual cycle, it may play a role in oocyte maturation, granulosa cell proliferation and death, and estradiol and progesterone production [105].

### 5.2. Leptin

Many authors are concordant on leptin playing a significant role in the onset and progression of puberty in humans. It is well known that congenital leptin deficiency, due to mutations in leptin gene or leptin receptor gene, cause early-onset obesity and absence of pubertal development [106]. At onset of puberty in healthy boys leptin concentrations rise by approximately 50% even before the increase in testosterone, LH, and FSH and then subsequently decline to baseline values [107–110].

In addition, nocturnal peaks in leptin secretion before puberty have been demonstrated in both rats and primates [111, 112]. All these data imply a role for leptin especially in the onset of puberty [113, 114].

Leptin concentrations have been shown to rise persistently during puberty also between Tanner stages III and IV. In addition, the same authors showed a significant decrease of soluble leptin receptor concentrations (SOB-R) and subsequently a continuous rise in free leptin index (FLI—ratio between leptin and SOB-R) which are more evident during Tanner stages I and II. A rapid FLI rise from Tanner stage I to II might be therefore implicated in the onset of puberty and thelarche in females. These observations suggest that FLI may be a better predictor of pubertal onset and sexual maturation compared to serum leptin concentrations alone, which conversely reflects more sensitively body composition and could therefore be a better predictor of forthcoming menarche [115]. Moreover, recent molecular and genetic data on mouse models support a permissive role for leptin in the normal progression of pubertal development [116]. Indeed, numerous studies have shown how leptin may contribute to hypothalamic-pituitary-gonadal regulation, both at the central and gonadal levels [117].

At the central level, leptin has been shown to have facilitatory effects on GnRH secretion; leptin increases pulsatile activity of GnRH neurons in the hypothalamus in different species and in both sexes [116].

Leptin acts both on glutamatergic ventral premammillary nucleus (PMV) neurons and on GABAergic AgRP neurons in the arcuate nucleus, with a stimulatory effect for the first ones and an inhibitory effect on the second ones. These groups of neurons are both linked to GnRH and/or kisspeptin neurons, thus modulating the production of GnRH [116]. Therefore, leptin may have a crucial role in the control of the hypothalamic-pituitary-gonadal axis through the modulation of these opposite signals according to the nutritional status of the body. It is important to underline that the absence of leptin signal in one of these neuronal groups does not, however, prevent sexual development, suggesting a possible compensating effect of one group of neurons on the other. These observations confirm the role of leptin as a facilitatory agent in sexual maturation and pubertal onset. In addition to its action on the hypothalamus, leptin has also been shown to control gonadal activity. Indeed, several authors have demonstrated the expression of leptin receptor in both male and female gonadal tissues; in particular, the leptin receptor has been found to be expressed in ovarian follicular cells, including granulosa, theca, and interstitial cells, and in Leydig cells [116, 117]. In vivo and in vitro studies showed how leptin reduces ovulation [118].

In the ovary, high leptin concentrations seem to have an inhibitory action on estradiol production and, therefore, block the maturation of dominant follicles and oocytes. Leptin also has a similar action in male gonadal tissue, with a dose-dependent inhibitory effect on testosterone production from Leydig cells as demonstrated in rats [119]. Therefore, leptin would have a bimodal action on the hypothalamic-pituitary-gonadal axis: at low concentrations, such as in starvation states, it would have a permissive threshold effect on the central neuronal circuits and, at high concentrations, on the contrary, as in obese subjects, it would have an inhibitory action on the gonads [118].

### 5.3. Resistin and Visfatin

No data are available to date as to resistin and visfatin in relationship with pubertal development.

Concerning gonadal function, there is only one study from Rak-Mardyła et al. in which the authors suggest a potential autocrine and/or paracrine role for visfatin in the regulation of estrous cycle and ovarian follicles development in porcine [120].

In Table 3 we detail the role of adipokines in puberty and pubertal development including key hormonal factors and molecules.

## 6. Adipokines in Growth and Pubertal Disorders

### 6.1. Adipokines and Premature Pubarche

Premature pubarche is classically defined as the development of pubic hair before the age of 8 years in females and 9 years in males. The most common cause of this phenomenon is a premature activation of the adrenal cortex, a process, known as premature adrenarche, characterized by an increased production of adrenal androgens, especially DHEA, DHEA-S, and androstenedione. The mechanisms underlying the development of premature adrenarche are still not well understood. Some authors have shown an association between premature pubarche and later development of insulin resistance, dyslipidemia, PCOS, and metabolic syndrome (especially in patients born small for gestational age); however, in most cases, it represents a variant of normal pubertal development [95, 121].

Adipokine patterns in girls with premature pubarche have been investigated, with few statistically significant data present in the literature and in some cases with conflicting results.

One study by Larqué et al. evaluated postprandial adiponectin response in girls with premature pubarche (PP) compared to controls; this work showed a significantly lower postprandial adiponectin area under the curve in PP girls, even after adjusting for BMI and age [122].

Teixeira et al. studied leptin concentrations in girls with premature pubarche, finding higher leptin levels compared to controls, independent of insulin and androgen concentrations [123].

We are not aware of any studies relating premature pubarche with resistin and visfatin.

### 6.2. Adipokines and Constitutional Delay of Growth and Puberty

Constitutional delay of growth and puberty (CDGP) is a condition characterized by a delay in the onset of pubertal development, representing a variant of the normal pubertal pattern without a defined endocrine abnormality. Data in the literature are overall still scarce, and basically only observational studies are available.

Leptin concentrations have been reported to be lower in adolescent boys with CDGP compared with regular and early maturers [124]. The authors further described a positive correlation between leptin, BMI, bone age, testicular volume, FSH, LH, and testosterone concentrations confirming previous findings.

Therefore, sexual immaturity in adolescents with CDGP seems to be characterized, at least in part, by decreased leptin concentrations [124].

### 6.3. Adipokines and Polycystic Ovarian Syndrome

Polycystic ovarian syndrome (PCOS) is one of the most common disorders in females, affecting approximately 15% of women during reproductive age [125]. It is classically characterized by three distinctive features: hyperandrogenism, ovarian dysfunction, and polycystic morphology pattern of ovaries on ultrasound scan [126]. A pivotal role in the pathophysiology of this syndrome is played by visceral adiposity and insulin resistance. Considering the importance of the adipokine milieu in modulating insulin sensitivity and hypothalamic-pituitary-gonadal axis, many authors have suggested a central role for adipokines in the development of hyperandrogenism and infertility in PCOS. Obese women are more prone to developing PCOS, and considering that these subjects show per se increased plasma concentrations of adipokines as leptin and lower concentrations of adiponectin, it is difficult to verify an independent effect of adipokines in PCOS. The aim of this section is to summarize the role of several adipokines in the pathogenesis of PCOS [127].

The role of adiponectin in polycystic ovary syndrome is still debated. Several studies have reported that adiponectin levels in women with PCOS are lower compared to non-PCOS controls with comparable BMI. In vitro, adiponectin has been shown to inhibit androgen production, key enzymes involved in androgen pathways, and LH receptor gene expression from theca cells [128]. Moreover, in bovine theca cells, the knockdown of adiponectin receptor genes determined an increase in androstenedione secretion [105].

These observations suggested that fat cell metabolism may be linked to ovarian steroidogenesis through adiponectin secretion and that disruption of adiponectin and its pathway may play an important part in the onset of hyperandrogenism in PCOS.

In addition, several authors have reported a correlation between lower adiponectin concentrations and the insulin insensitivity observed in women with PCOS compared with controls [129].

Leptin has been thought to play a role in the onset of ovarian dysfunction in obese subjects, as previously mentioned; hyperleptinemia might interfere with estrogen production and maturation of dominant follicles [130]. However, most authors agree that there are no significant differences in leptin levels in women with PCOS compared with controls, even after correction for weight and BMI [131].  Table 4 summarizes the main findings published in the literature concerning the relationship between adiponectin and leptin and onset of PCOS.

The contribution of other adipokines, such as resistin and visfatin, to the pathophysiology of PCOS has been largely debated, but there is no conclusive evidence in the literature for a significant role.

Some authors have studied resistin serum concentrations in PCOS, but the results of these studies at this time remain conflicting [125, 131].

Patients with type 2 diabetes, women with gestational diabetes, and women with PCOS tend to have higher plasma visfatin concentrations compared with controls, which would confirm its role in the determinism of insulin resistance and thus a potential role in the control of ovarian steroidogenesis.

## 7. Conclusions

Recent studies have focused on the production of adipokines not only by the adipose tissue but also by other organs such as placenta, ovaries, peripheral-blood mononuclear cells, liver, muscle, kidney, heart, and bone marrow, introducing the concept of a more pleiotropic action of these hormones. The regulation of insulin sensitivity remains, however, central to their action, and tightly related to pre- and post-natal longitudinal growth and weight increase, pubertal development, and pubertal disorders.

Many of these aspects, however, remain not entirely understood and need further investigation.

Summarizing the main findings to date consists in adiponectin being an upregulator of insulin and energy balance. Leptin mediates mainly food intake, liver glucose production, gonadotropin secretion, suppression of lipogenesis in adipose tissue, and modulation of immune responses. Circulating levels of leptin are related to adipose mass. Resistin is likely to have an inhibitory effect on adipose conversion, acting as a feedback regulator of adipogenesis and adipose tissue. Recently, visfatin has been described as a protein with insulin-like functions.

There is evidence that these adipokines are important in placental and fetal growth, programming of insulin sensitivity, and present relationships with birth weight.

Pubertal development is physiologically characterized by an increase in adipose mass and changes in adipose tissue distribution. Many authors are concordant on a significant role of leptin in pubertal initiation and progression in humans. In healthy lean males, but not in females, adiponectin concentrations significantly reduce during puberty, with lower adiponectin levels in adolescent boys compared to girls.

Timing and progression of puberty is related to being born small or appropriate for gestational age and preterm or full-term, and size at birth is related mainly to premature pubarche, an earlier onset of pubertal development and menarche, and subsequent development of PCOS.

Some data show that adipokines concentrations are different in most disorders of puberty, although at present data they are not definitive and not always consistent. Altogether, data are yet scarce and further studies are warranted.

## Figures and Tables

**Table 1:** Site of production and relationship of adipokines with inflammation and metabolic and reproductive function.

Adipokine	Site of production	Relationship with inflammation	Relationship with metabolic functions	Relationship with reproductive function
Adiponectin	Adipocytes Placenta Osteoblasts Cardiomyocytes	Anti-inflammatory functions [30–33]	Fatty acid oxidation [22] Insulin sensitivity [24] Glucose metabolism [24–29]	Pubertal onset and regulation [104, 105]

Leptin	Adipose tissue Placenta	Regulation of immune response [34]	Regulation of food intake, lipid and glucose metabolism [37, 38]	Regulation of gonadotropin secretion [39–41]

Resistin	Peripheral-blood mononuclear cells Placenta	Regulation of inflammatory response [43, 48, 50]	Insulin resistance [44–46]	Ovarian function and PCOS [55]

Visfatin	Adipose tissue Liver Muscle Kidney Heart Bone marrow Placenta	Regulation of inflammatory responses [51]	Insulin resistance Glucose metabolism [52]	Ovarian function and PCOS [55]

**Table 2:** Role of adipokines in fetal and postnatal growth and in the onset of gestational metabolic disturbances.

Adipokine	Fetal growth and birth weight	Postnatal catch-up growth	Gestational diabetes
Adiponectin	Yes [8, 63]	Yes [68]	Yes [64]
Leptin	Yes [71–78]	Not described	Yes [79]
Resistin	Controversial [83, 84]	Not described	Controversial [83, 85, 86]
Visfatin	Controversial [87]	Not described	Controversial [88]

**Table 3:** Role of adipokines in puberty.

Adipokine	GnRH, LH, and FSH	Estradiol (E2) and testosterone (T)
Adiponectin	AdipoR2 is regulated by the LH receptor function via a cAMP dependent mechanism [132].	T inhibits secretion from adipocytes. E2 controls oocyte maturation and granulosa cells' cycle [104, 105].

Leptin	Rises before LH and FSH surge and subsequently declines promoting sexual maturation and pubertal onset. Induces secretion of GnRH and increases pulsatile activity [116, 117].	Rises before T increase and subsequently declines. Reduces ovulation inhibiting E2 and T production acting on Leydig cells [118, 119].

Resistin	Not described	Not described

Visfatin	Not described	Not described

**Table 4:** Main findings published in the literature concerning the relationship between adiponectin, leptin and onset of PCOS.

Author	Adipokine studied	Type of study	In vivo/species	Main conclusions
Comim et al. [105]	Adiponectin	Case control	In vitro/humans and bovine	There is strong evidence for a direct link between fat cell metabolism and ovarian steroidogenesis, suggesting that disruption of adiponectin and/or its receptors plays a key role in pathogenesis of hyperandrogenism in PCOS.

Toulis et al. [129]	Adiponectin	Meta analysis	In vivo/humans	Adiponectin levels seem to be lower in women with PCOS compared with non-PCOS controls. Low levels of adiponectin in PCOS are probably related to insulin resistance but not to testosterone levels. Total adiponectin should not be used as a biomarker of PCOS severity. Further investigations are needed to understand the role of high molecular weight adiponectin levels in PCOS.

Svendsen et al. [131]	Adiponectin, leptin	Cross-sectional	In vivo/humans	PCOS does not appear to have an independent effect on the adipose expression of leptin, adiponectin, and IL-6 or the circulating adipocytokines.

Li et al. [127]	Leptin	Case control	In vivo/humans	A significant association was found between the Pro1019Pro in LEPR gene and PCOS, and a highly significant association was found between the Gln223Arg in LEPR gene and PCOS.
